# Hyperhomocysteinemia and Low Folate and Vitamin B12 Are Associated with Vascular Dysfunction and Impaired Nitric Oxide Sensitivity in Morbidly Obese Patients

**DOI:** 10.3390/nu12072014

**Published:** 2020-07-07

**Authors:** Mohamed Haloul, Smita Jagdish Vinjamuri, Dina Naquiallah, Mohammed Imaduddin Mirza, Maryam Qureshi, Chandra Hassan, Mario Masrur, Francesco M. Bianco, Patrice Frederick, Giulianotti P. Cristoforo, Antonio Gangemi, Mohamed M. Ali, Shane A. Phillips, Abeer M. Mahmoud

**Affiliations:** 1 Division of Endocrinology, Diabetes, and Metabolism, Department of Medicine, College of Medicine, University of Illinois at Chicago, Chicago, IL 60612, USA; haloul57@uic.edu (M.H.); svinja2@uic.edu (S.J.V.); dnaqui2@uic.edu (D.N.); mmirza24@uic.edu (M.I.M.); mshafi1@uic.edu (M.Q.); shanep@uic.edu (S.A.P.); 2 Clinical Pathology Laboratory, Children’s Cancer Hospital Egypt, Cairo 57357, Egypt; 3 Departments of Surgery, College of Medicine, University of Illinois at Chicago, Chicago, IL 60612, USA; chandrar@uic.edu (C.H.); mmasrur@uic.edu (M.M.); biancofm@uic.edu (F.M.B.); pfrede1@uic.edu (P.F.); piercg@uic.edu (G.P.C.); agangemi@uic.edu (A.G.); 4 Department of Physical Therapy, College of Applied Health Sciences, University of Illinois at Chicago, Chicago, IL 60612, USA; mali37@uic.edu; 5 Integrative Physiology Laboratory, College of Applied Health Sciences, University of Illinois at Chicago, Chicago, IL 60612, USA

**Keywords:** homocysteine, folate, vitamin B12, obesity, vascular dysfunction, bariatric surgery, nitric oxide

## Abstract

There is a high prevalence of hyperhomocysteinemia that has been linked to high cardiovascular risk in obese individuals and could be attributed to poor nutritional status of folate and vitamin B12. We sought to examine the association between blood homocysteine (Hcy) folate, and vitamin B12 levels and vascular dysfunction in morbidly obese adults using novel ex vivo flow-induced dilation (FID) measurements of isolated adipose tissue arterioles. Brachial artery flow-mediated dilation (FMD) was also measured. Subcutaneous and visceral adipose tissue biopsies were obtained from morbidly obese individuals and non-obese controls. Resistance arterioles were isolated in which FID, acetylcholine-induced dilation (AChID), and nitric oxide (NO) production were measured in the absence or presence of the NO synthase inhibitor, L-NAME, Hcy, or the superoxide dismutase mimetic, TEMPOL. Our results demonstrated that plasma Hcy concentrations were significantly higher, while folate, vitamin B12, and NO were significantly lower in obese subjects compared to controls. Hcy concentrations correlated positively with BMI, fat %, and insulin levels but not with folate or vitamin B12. Brachial and arteriolar vasodilation were lower in obese subjects, positively correlated with folate and vitamin B12, and inversely correlated with Hcy. Arteriolar NO measurements and sensitivity to L-NAME were lower in obese subjects compared to controls. Finally, Hcy incubation reduced arteriolar FID and NO sensitivity, an effect that was abolished by TEMPOL. In conclusion, these data suggest that high concentrations of plasma Hcy and low concentrations of folate and vitamin B12 could be independent predictors of vascular dysfunction in morbidly obese individuals.

## 1. Introduction

Obesity is a major public health concern that affects more than one-third of the population and increases the risk of other health problems, including metabolic and cardiovascular diseases [1]. Several factors contribute to the increasing trend in obesity, including genetic predisposition and lack of physical activity, yet the most predominant factor is excess caloric intake. Despite overeating, obese individuals have a relatively high incidence of micronutrient deficiencies [2]. Some of these micronutrients act as cofactors in critical biological pathways in the body, such as energy metabolism and immune function. One of these vital biological processes that are regulated by the bioavailability of micronutrients, namely folate and other B vitamins, is One-Carbon metabolism [3].

In the “One-Carbon metabolism”, dietary folate is converted to dihydrofolate (DHF) then to tetrahydrofolate (THF) that, in turn, is converted to 5-methyl THF via a series of enzymatic reactions cofactored by vitamins B6 and B12, and betaine. The final product, 5-methyl THF, donates its methyl group to homocysteine (Hcy) to produce methionine, which is critical for the formation of the ultimate methyl donor, S-Adenosylmethionine (SAM). The latter is metabolized to S-adenosylhomocysteine (SAH), which could be reversibly converted to Hcy via the enzyme SAH hydrolase. The fate of Hcy is through re-methylation to methionine via the folate-dependent pathway or transsulfuration to cystathionine, via cystathionine β-synthase [3].

Hyperhomocysteinemia refers to increased plasma levels of Hcy and has been classified into three categories: mild (15–30 µmol/L), moderate (30–100 µmol/L), and severe (100 µmol/L) [4]. Several pathological conditions could cause hyperhomocysteinemia, including dysfunction of the enzymes that are associated with homocysteine biosynthesis and metabolism such as methyl-THF reductase and cystathionine β-synthase or deficiency in cofactors such as folate and vitamins B2, B6, and B12 [5]. Hyperhomocysteinemia is considered an established, independent risk factor for cardiovascular disease (CVD), including atherosclerosis and coronary artery disease [6,7]. Despite this growing evidence of the role of hyperhomocysteinemia in vascular dysfunction, its effect and the mechanistic drive of this effect on human microvasculature are largely unexplored. In the current study, we aimed to investigate the hypothesis that hyperhomocysteinemia is associated with microvascular dysfunction in morbidly obese adults. Using the proposed ex vivo system in this study, we were able to explore the differential responses to vasoactive mediators in arterioles preconditioned with hyperhomocysteinemia compared to those isolated from subjects with normal Hcy levels. Moreover, we measured other variables that were previously proposed to contribute to hyperhomocysteinemia such as folate, vitamin B12, and insulin levels, as well as alcohol intake [5]. 

## 2. Methods

### 2.1. Human Participants

Subjects were 40 obese adults and 40 non-obese controls who underwent bariatric surgeries and elective surgeries (non-inflamed hernias and cholecystectomies), respectively, at the University of Illinois Medical Center. Inclusion criteria included age from 21 to 49 years old, a BMI higher than 35 kg/m^2^ for the obese group and less than 30 kg/m^2^ for the controls, and the absence of significant chronic or inflammatory disease that may modify vascular outcomes. Excluded subjects included those above 50 years old, postmenopausal, and pregnant women, smokers, subjects with a history of previous bariatric surgery, and individuals with current cardiac, hepatic, or renal disease, malignancy, or acute or chronic inflammatory conditions. Evaluating subjects for eligibility criteria took place before the first data collection clinical visit. The study team informed the eligible subjects about the study specifics and provided them with a written informed consent. All protocols and methods that were used in this study followed the regulations established by the most recent revision of the Declaration of Helsinki and were approved by the University of Illinois Institutional Review Board. During subject’s clinical visit, blood samples and anthropometric/body composition measurements were collected, as well as brachial artery ultrasound imaging. In addition, information about alcohol administration and the intake of supplements that contain folate or vitamin B12 were obtained via questionnaires. For alcohol consumption, subjects were classified into (1) light drinkers, those who consume alcohol less than one time/month with less than 5 drinks/time, 1–3 times/month with less than 3 drinks/time or 1–2 times/week with less than 2 drinks/time; (2) moderate drinkers, those who consume alcohol 1–3 times/month with 3–4 drinks/time, 1–2 times/week with 2–4 drinks/time, or 3–6 times/week with less than 2 drinks/time; and (3) heavy drinkers, those who consume alcohol at any quantity and/or frequency that is more than moderate drinkers. On the day of bariatric surgery, adipose tissue samples, both visceral (VAT) and subcutaneous (SAT) were provided to us by the surgeon and put in ice-cold HEPES buffer for transfer to the laboratory for dissection and isolation of microvessels.

### 2.2. Physical Measurements and Body Composition

Physical characteristics including body mass, body mass index (BMI), and waist circumference were assessed. Dual X-ray absorptiometry (DXA; iDXA, General Electric Inc., Boston, MA, USA) was used to quantify lean, total fat, and visceral fat mass. All subjects had a single scan performed on Lunar iDXA. Subjects were positioned on the scanner following the operator’s manual, and all women had confirmed negative pregnancy tests before scanning.

### 2.3. Cardiometabolic Measurements

Biochemical measurements of lipid profile and glucose metabolism were performed in fasting blood samples. Plasma glucose concentration was measured using a standard glucometer (LifeScan). Insulin was measured via a highly sensitive Insulin ELISA kit, ENZ-KIT141-0001 (Enzo Life Sciences, Inc., Farmingdale, NY, USA) following the producer’s protocol. To assess insulin resistance, we used the homeostasis model assessment formula, HOMA-IR which is calculated by dividing the product of fasting insulin (µU/L) and fasting glucose (nmol/L) by 22.5, as previously described [8]. Triglycerides, total cholesterol, high-density lipoproteins (HDL), and direct low-density lipoproteins (LDL) were measured on a Hitachi 911 analyzer using enzymatic assays from Roche Diagnostics (Indianapolis, IN, USA) and following to the manufacturer’s specification.

### 2.4. Plasma Hcy, Folate, and Vitamin B12

Total plasma Hcy levels were measured using a Hcy ELISA Kit (Cell Biolabs Inc., San Diego, CA, USA), following the manufacturer’s guidelines. Briefly, plasma samples were diluted 2X and incubated in the Hcy conjugate coated plate for 10 min at room temperature. Then, the primary anti-Hcy antibody was added and incubated for one hour, followed by washing steps and the addition of the secondary antibody. Finally, the provided substrate was incubated for 30 min after which the reaction was stopped using the provided stop solution. Absorbance was measured via iMark Microplate Reader (BioRad, Hercules, CA, USA) using 450 nm as the primary wavelength. Plasma levels of folate and vitamin B12 were assessed via Elecsys Folate III (Roche Diagnostics; Indianapolis, IN, USA) approach that utilizes a competitive assay principle via natural, specific folate binding protein and specific intrinsic factor for vitamin B12, respectively.

### 2.5. Serum NO Measurements

Serum concentrations of nitrate and nitrite, stable NO metabolites, were measured using the Griess reaction (Cayman Chemicals, Ann Arbor, MI) as we previously described [9]. Briefly, serum samples were ultra-filtered through 10 KDa molecular weight cut-off filters from Millipore (Burlington, MA, USA). In filtered samples, nitrate reductase converted nitrates into nitrites, which in turn is converted into a dark purple azo compound when Griess reagents were supplied. Absorbance was measured at 540 nm using iMark Microplate Reader. A nitrate standard curve was included in the experiment and used to calculate nitrate concentrations in samples.

### 2.6. Brachial Artery Flow-Mediated Dilation (FMD)

**Figure 1:**
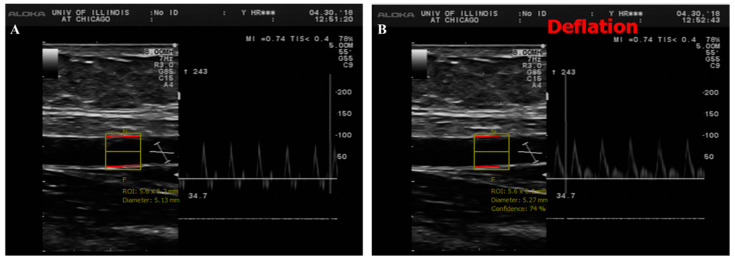
Duplex B-mode/pulsed wave Doppler (PWD) ultrasound of brachial artery flow-mediated dilation (FMD). This figure shows a long axis scan of brachial artery with simultaneous blood velocity profile by pulsed wave Doppler before (**A**) and after cuff deflation (**B**). For offline image analyses, a representative section of brachial artery is selected for an automated measurement of diameter. Baseline diameter (BSL) was averaged from a serial of recorded frames before cuff deflation and the maximum diameter was measured after cuff deflation during reactive hyperemia (RH).

Brachial imaging was performed on Hitachi Prosound Alpha 7 (Hitachi Aloka Medical America, Wallingford, CT, USA) using a linear probe placed about 5 cm above the antecubital fossa of the left arm, abducted at about 90 degrees relative to the body torso. After a 1-min baseline imaging (BSL), an inflatable blood pressure cuff was wrapped around the right mid-forearm and inflated up to 200 to 220 mmHg for 5 min. After cuff deflation, a 300-s long video sequence at three frames/second was recorded using a video grabber for offline measurement. The imaging protocol involved acquiring at least 60 s of BSL diameter before inflating the cuff and 300 s for the reactive hyperemia (RH) event induced by the cuff deflation. Blood flow velocity was acquired simultaneously using pulsed wave Doppler. Brachial Analyzer software (Medical Imaging Applications LLC, Coralville, IA, USA) was used to analyze the brachial artery diameter (Figure 1). Percent changes in FMD were calculated as follows [%FMD = (RH diameter in mm – BSL diameter in mm/BSL diameter in mm) × 100].

### 2.7. Microvascular Preparations

Adipose tissues were cleaned of excess connective tissue and carefully dissected to isolate visceral and subcutaneous resistance arterioles. Isolated arterioles were used to measure microvascular reactivity as we previously described [9,10,11]. Briefly, both SAT and VAT arterioles were cannulated and mounted in an organ perfusion chamber where arteriolar ends were secured using 10-0 nylon Ethilon monofilament suture. The whole setup was then placed on the stage of an inverted microscope to which a video camera is attached. Images were displayed on a video monitor and the internal arteriolar diameter was measured via a video measuring device (model VIA-100; Boeckeler, Madison, WI, USA). To maintain arterioles under physiological conditions, warm physiological salt solution (Krebs buffer) was continuously perfused inside the organ chamber. Krebs buffer consisted of the following ingredients in mmol/L: 123 NaCl, 4.4 KCL, 20 NaHCO_3_, 2.5 CaCl_2_, 1.2 KH_2_PO_4_, 1.2 MgSO_4_, and 11 glucose. The temperature of the buffer was maintained at 37 °C, and the pH was adjusted to 7.4. The buffer was also provided with a mixture of air that contains 21% O_2_, 5% CO_2_, and 74% N_2_. The arteriolar ends were connected to physiological buffer-containing reservoirs that were used to adjust the intraluminal pressure gradient (10–100 cmH_2_O) [12].

### 2.8. Flow-Induced Dilation (FID) Measurements

Endothelin-1 (Peninsula, San Carlos, CA, USA) was used to pre-constrict cannulated arterioles and those that demonstrated less than 30% constriction compared to baseline were excluded from the study since they are mostly damaged during processing [9,10,11,13,14,15]. Internal diameters of cannulated arterioles were measured at baseline conditions and during continuing increases of the intraluminal pressure gradient (10–100 cmH_2_O), acetylcholine concentration (ACh; 10^−9^–10^−4^ M) [10], or the NO donor, sodium nitroprusside (SNP; 10^−9^–10^−4^ M). Measurements were repeated after incubations with the endothelial nitric oxide synthase inhibitor L-NAME (10^−4^ M). All treatments were applied for 30 min followed by measuring FID and acetylcholine-induced dilation (AchID). In a subset of subjects (ten obese and ten controls), arterioles were incubated with 100 µM of Hcy for 180 min [16] with and without L-NAME or the superoxide dismutase mimetic, 4-Hydroxy-TEMPO (TEMPOL, 10^−5^ M). Maximum dilation of arterioles was assessed at the end of each experiment using the antispasmodic, Papaverine at a concentration of 10^−4^ M. Arteriolar vasodilation was calculated as the percentage change in arteriolar diameter following different treatments relative to the diameter after ET-1-induced constriction. All the above-mentioned chemicals except endothelin-1 were acquired from Sigma Aldrich (St. Louis, MO, USA).

### 2.9. Measurements of Arteriolar NO and Reactive Oxygen Species (ROS)

Nitric oxide and ROS generation in the adipose tissue-isolated arterioles were measured as we previously described [17] using NO Detection Kit (Enzo Life Sciences, Inc., Farmingdale, NY, USA) and ROS green fluorescent detection reagent, 2′,7′-dichlorodihydrofluorescein diacetate (H_2_DCFDA) (Thermo Fisher Scientific). Flow-induced generation of nitric oxide and ROS were detected in cannulated arterioles that were kept at 37 °C in Krebs solution that was supplied by a 20% O_2_/5% CO_2_ gas mixture. Vessels were maintained at an equilibration pressure of 60 cm H_2_O for one hour. Vessels incubated in 10^−5^ mol/L ACh or 10^−3^ mol/L BSO served as positive controls for NO and superoxide generation, respectively. Vessels were stained, mounted on microscopic coverslips, and imaged via fluorescence microscopy (Eclipse TE 2000, Nikon, Japan) at wavelengths of 650/670 nm and 495/527 nm for NO and ROS detection, respectively. All procedures for incubation, staining, and detection were consistent among all experiments and treatment conditions. Fluorescence intensities in the developed images were measured and expressed in arbitrary units using NIH Image J software (NIH, Bethesda, MD, USA).

### 2.10. Statistical Analyses

All findings were reported as the mean ± standard error and a *p* value less of than 0.05 was considered statistically significant. Fluorescent intensities were analyzed using NIH Image J software (NIH, Bethesda, MD, USA) after correcting for background autofluorescence. Physical features, cardiometabolic parameters, and vascular measurements were assessed using Student’s unpaired *t*-test for between group comparisons. Statistically significant linear relationship between continuous variables were tested using a bivariate Pearson Correlation. Arteriolar vasodilation was presented as a percentage increase in diameter in response to different treatment conditions relative to the pre-constricted state. Multivariate regression analysis was run to predict vasodilation (FID and AchID) from other independent variables. Analyses were conducted using SPSS statistical software (version 26.0; SPSS Inc., Chicago, IL, USA).

## 3. Results

### 3.1. Physical and Cardiometabolic Parameters

Physical characteristics, including age, gender, body weight, BMI, waist circumference, fat percentage, and cardiometabolic risk factors including blood pressure, heart rate, lipid profile, and glucose metabolism are displayed in Table 1. Body weight, waist circumference, BMI, and fat percentage were significantly higher in obese compared to non-obese subjects (*p* < 10^−10^). Moreover, heart rate, systolic and diastolic blood pressure were higher in the obese group (*p* < 0.01). Although the average fasting blood glucose and HbAlc were not statistically different between the two groups, the average fasting plasma insulin and HOMA-IR were lower in the control group compared to obese subjects by 43% and 52%, respectively. Total cholesterol, LDL, and triglycerides did not differ between the groups; however, the average HDL level was 30% higher (*p* = 0.0004) in the non-obese compared to obese subjects. Biomarkers of inflammation namely interleukin 6 (IL6), IL8, and C-reactive protein (CRP) were 3.2-fold, 63%, and 4.4-fold higher, respectively, in the obese subject compared to the non-obese controls. These inflammatory biomarkers correlated negatively with vascular functions that were measured via brachial artery FMD or arteriolar FID.

### 3.2. Plasma Hcy, Folate, and Vitamin B12 and Serum NO Measurements

**Figure 2:**
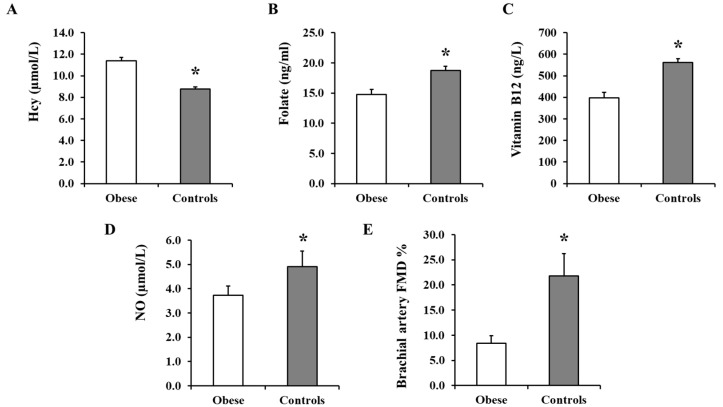
Plasma Hcy, folate, vitamin B12, and NO concentrations and brachial artery FMD measurements. Plasma from obese subjects (*n* = 40) and non-obese controls (*n* = 40) were analyzed for Hcy (**A**), folate (**B**), and vitamin B12 (**C**) using specific ELISA assays and for nitrates + nitrites (NO metabolites) using the Griess chemical reaction assay (**D**). Percentage of brachial artery FMD was calculated by subtracting the mean baseline diameter from the largest mean values obtained after cuff deflation in obese subjects (*n* = 40) and non-obese controls (*n* = 40) (**E**). All measurements are presented as means ± standard error (SE). * (*p* < 0.05) for comparing obese subjects with controls.

The average total plasma Hcy was found to be significantly higher in the obese group (1.5 ± 0.04 µg/mL; equivalent to 11.4 ± 0.3 µmol/L) compared to the non-obese group (1.2 ± 0.03 µg/mL; equivalent to 8.7 ± 0.2 µmol/L; *p* < 0.0001) (Figure 2A). Plasma Hcy correlated positively with body weight (*r* = 0.41, *p* = 0.004), waist circumference (*r* = 0.43, *p* = 0.014), BMI (*r* = 0.45, *p* = 0.002), and fat percentage (*r* = 0.41, *p* = 0.016), and negatively with lean percentage (*r* = −0.40, *p* = 0.017). Interestingly, plasma Hcy correlated significantly with fasting insulin levels (*r* = 0.47, *p* = 0.001) and when obese subjects were divided into hyperinsulinemic (>9 µU/mL) and normoinsulinemic (<9 µU/mL) groups, the hyperinsulinemic group had a higher level of Hcy (12.9 ± 0.2 µmol/L) compared to the normoinsulinemic one (8.9 ± 0.4 µmol/L, *p* < 0.01). The average of plasma folate was ~27% higher in the non-obese controls compared to obese subjects (18.7 ± 0.7 ng/mL vs. 14.8 ± 0.8 ng/mL; *p* = 0.0004) (Figure 2B). Plasma folate correlated negatively with body weight (*r* = −0.36, *p* < 0.0001), VAT mass (*r* = −0.49, *p* = 0.008), and fasting plasma insulin (*r* = −0.21, *p* = 0.044). Similarly, vitamin B12 levels were ~41% higher in the non-obese compared to obese subjects (561.8 ± 17.9 ng/L vs. 397.5 ± 26.3 ng/L; *p* < 0.0001) (Figure 2C) and correlated negatively with body weight (*r* = −0.39, *p* < 0.0001), total fat mass (*r* = −0.41, *p* = 0.004), fasting plasma insulin (*r* = −0.30, *p* = 0.005), and HOMA-IR (*r* = −0.23, *p* = 0.028). Finally, serum levels of nitrates and nitrites, as a surrogate marker of NO bioavailability, were ~32% higher in the non-obese compared to obese subjects (4.9 ± 0.6 µmol/L vs. 3.7 ± 0.4 µmol/L; *p* = 0.047) (Figure 2D).

### 3.3. Brachial Artery FMD

Baseline arterial diameter was not statistically different between the two groups, obese (6.1 ± 0.6) and non-obese ((5.2 ± 0.2), *p* = 0.619). The %FMD, calculated as described in the methods, was 1.6-fold lower in the obese subjects compared to the non-obese controls (*p* = 0.017; Figure 2E). Percentage FMD correlated positively with lean % (*r* = 0.71, *p* < 0.0001), folate (*r* = 0.32, *p* = 0.030), and vitamin B12 (*r* = 0.67, *p* < 0.0001) and negatively with body weight (*r* = −0.87, *p* < 0.0001), BMI (*r* = −0.89, *p* < 0.0001), total fat % (*r* = −0.57, *p* = 0.001), VAT mass (*r* = −0.77, *p* < 0.0001), Hcy (*r* = −0.47, *p* = 0.007), and HOMA-IR (*r* = −0.32, *p* = 0.024).. Furthermore, there were significant direct correlations between %FMD and arteriolar FID (at Δ 60, *r* = 0.43, *p* = 0.004) and AchID (at 10^−5^ mole/L, *r* = 0.51, *p* = 0.001) among the subjects.

### 3.4. Arteriolar Vasoreactivity and NO and ROS Production

**Figure 3:**
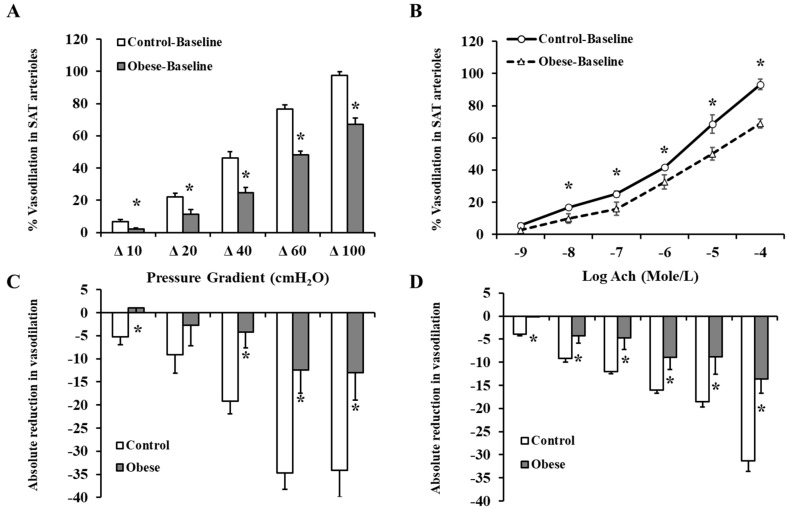
FID and acetylcholine-induced dilation (AchID) in SAT isolated resistance arterioles. FID measurements in SAT arterioles isolated from obese (*n* = 40) and non-obese (*n* = 40) subjects corresponding to increasing intraluminal pressure gradients of 10–100 cmH_2_O (**A**). AchID measurements in SAT arterioles corresponding to increasing concentrations of Ach (10^−9^ to 10^−4^ M) (**B**). Absolute reduction in FID in response to eNOS inhibition via L-NAME (10^−4^ M) (**C**). Absolute reduction in AchID in response to eNOS inhibition via L-NAME (**D**). All measurements are presented as means ± standard error (SE). * (*p* < 0.05) for comparing obese subjects with controls.

**Figure 4:**
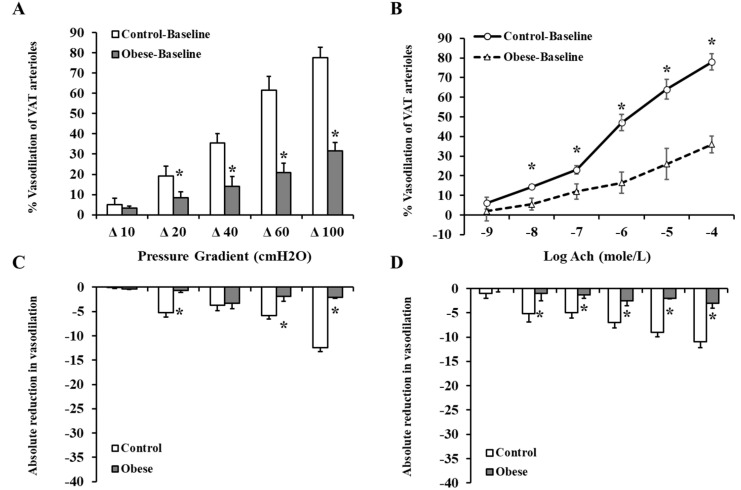
FID and AChID in VAT isolated resistance arterioles. FID measurements in VAT arterioles isolated from obese (*n* = 40) and non-obese (*n* = 40) subjects corresponding to increasing intraluminal pressure gradients of 10–100 cmH_2_O (**A**). AchID measurements in VAT arterioles corresponding to increasing concentrations of Ach (10^−9^ to 10^−4^ M) (**B**). Absolute reduction in FID in response to eNOS inhibition via L-NAME (10^−4^ M) (**C**). Absolute reduction in AchID in response to eNOS inhibition via L-NAME (**D**). All measurements are presented as means ± standard error (SE). * (*p* < 0.05) for comparing obese subjects with controls.

Figure 3 shows the response of isolated SAT arterioles to increasing the intraluminal pressure gradient (Δ 10–Δ 100 cmH_2_O). Arteriolar FID was higher in the non-obese compared to obese subjects across all pressure gradients. The maximum dilation (MD) at ∆ 60 cmH_2_O, which corresponds to physiological shear stress, was 60% higher in the controls (Figure 3A). Similar results were obtained in response to Ach (Figure 3B). The reduction in FID and AchID observed in SAT arterioles in response to endothelial nitric oxide synthase (eNOS) inhibition by L-NAME (Figure 3C,D) were of a higher magnitude in the controls compared to obese subjects (1.8 fold higher at ∆ 60 cmH_2_O, *p* < 0.0001). The observed low sensitivity of SAT arterioles to NO inhibition in obese subjects might indicate a disruption in the NO-dependent vasodilation mechanism in this group. Similar patterns of differences in the FID and AchID between obese and non-obese subjects were observed in the VAT arterioles (Figure 4A,B). The response of VAT arterioles to NO inhibition via L-NAME was less than that seen in SAT arterioles in both the obese (reduction in % vasodilation by 35 in SAT vs. 6 in VAT at ∆ 60 cmH_2_O) and non-obese (reduction in % vasodilation by 12 in SAT vs. 2 in VAT at ∆ 60 cmH_2_O) groups, indicating lower sensitivity of VAT arterioles to NO-induced vasoreactivity (Figure 4C,D). Furthermore, arteriolar FID and AchID were lower in the VAT compared to SAT arterioles in the non-obese (24% lower at ∆ 60 cmH_2_O, *p* < 0.01) and obese subjects (1.3 fold lower at ∆ 60 cmH_2_O, *p* < 0.0001); yet, the magnitude of reduction was much higher in the latter group across all pressure gradients and Ach doses. Baseline FID and AchID in SAT and VAT arterioles correlated significantly with anthropometric and cardiometabolic risk factors, as shown in Table 2 (only FID is shown). Using multivariate regression analysis, Hcy, folate, and vitamin B12 were independent predictive factors for the FID and AchID in both SAT and VAT arterioles. The magnitude of FID reductions following L-NAME incubation, which reflects NO sensitivity correlated negatively with weight, waist circumference, BMI, DEXA-estimated fat% and VAT mass, systolic blood pressure, HbA1c, fasting plasma insulin, HOMA-IR, triglycerides, and Hcy and positively with DEXA-estimated lean%, HDL, NO, folate, and vitamin B12 (Table 3). These correlations were more significant in SAT arterioles compared to VAT arterioles, indicating a higher NO sensitivity in the former.

**Figure 5:**
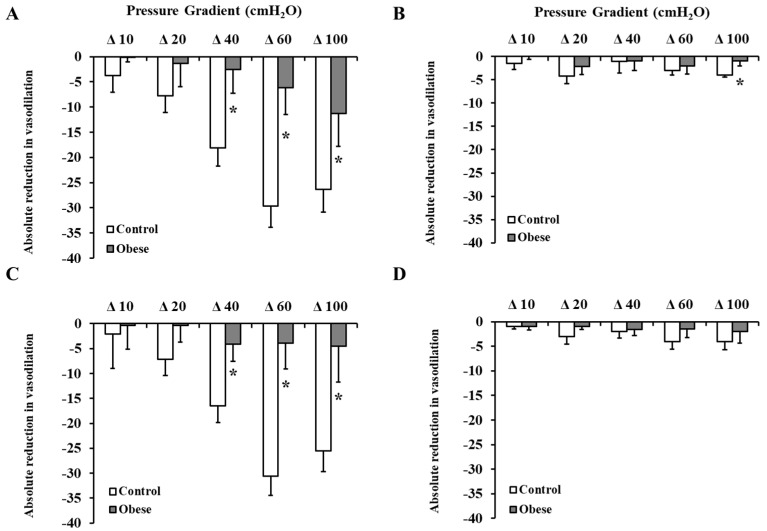
Effect of Hcy incubation on baseline FID and L-NAME-mediated reduction in FID. SAT (**A**,**B**) and VAT (**C**,**D**) isolated arterioles from obese subjects (*n* = 10) and non-obese controls (*n* = 10) were incubated in100 µM of Hcy for 180 min followed by measuring FID with and without eNOS inhibition via L-NAME (10^−4^ M). Charts (**A**,**C**) present absolute reductions in FID in Hcy preconditioned arterioles compared to corresponding unconditioned arterioles. Charts (**B**,**D**) present absolute reduction in FID caused by L-NAME in Hcy preconditioned arterioles compared with baseline FID after Hcy incubation. All measurements are presented as means ± standard error (SE). * (*p* < 0.05) for comparing obese subjects with controls.

**Figure 6:**
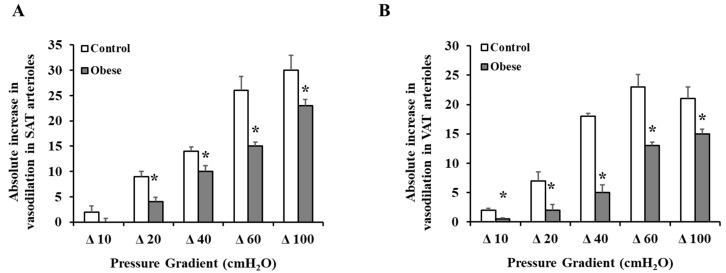
Effect of TEMPOL on restoring FID in Hcy preconditioned arterioles. Isolated arterioles from obese subjects (*n* = 10) and non-obese controls (*n* = 10) were incubated in100 µM of Hcy and the superoxide dismutase mimetic, TEMPOL (10^−5^ M) for 180 min followed by measuring the FID. Charts (**A**) and (**B**) present the absolute increase in FID in response to combined incubation with Hcy and TEMPOL relative to Hcy alone in SAT and VAT arterioles, respectively. All measurements are presented as means ± standard error (SE). * (*p* < 0.05) for comparing obese subjects with controls.

**Figure 7:**
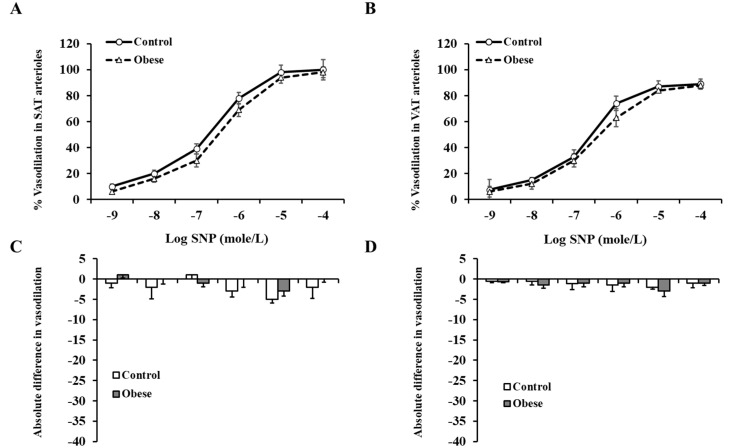
Endothelium-independent vasodilation in SAT and VAT isolated arterioles. The intraluminal diameter of SAT (**A**) and VAT (**B**) isolated arterioles was measured in response to increasing concentrations of SNP (10^−9^−10^−4^ M) in obese subjects (*n* = 40) and non-obese controls (*n* = 40). The absolute difference in SNP-induced vasodilation between Hcy-preconditioned and unconditioned SAT (**C**) and VAT (**D**) arterioles in obese subjects (*n* = 10) and non-obese controls (*n* = 10). All measurements are presented as means ± standard error (SE).

Exogenous incubation with Hcy reduced arteriolar vasodilation in both SAT (Figure 5A) and VAT (Figure 5C) arterioles. These reductions were of a higher magnitude in the controls compared to obese subjects. At ∆ 60 cm H_2_O, the average absolute reduction in % vasodilation was 30 in control SAT arterioles and 6 in obese SAT arterioles. Similar patterns were obtained in VAT arterioles. These findings could be explained by the higher baseline FID measurements in controls compared to obese subjects. These high measurements provide a chance for a more perceptible magnitude of reduction in the control arterioles in response to Hcy. In Hcy-preconditioned arterioles, L-NAME-mediated FID reductions were of very low magnitude in obese and non-obese subjects in both SAT (Figure 5B) and VAT (Figure 5D) arterioles. The observed low sensitivity of Hcy-preconditioned arterioles to NO inhibition might indicate a disruption in the NO-dependent vasodilation mechanism in these arterioles following incubation with Hcy. Vessels that were incubated with Hcy and TEMPOL, the superoxide dismutase mimetic, had higher FID measurements compared to Hcy alone in both obese and control subjects (Figure 6 A,B). For example, When TEMPOL was combined with Hcy, FID in SAT arterioles at ∆ 60 cm H_2_O pressure increased by 55% in controls and 36% in obese subjects compared to Hcy only. Similarly, FID in VAT arterioles at ∆ 60 cm H_2_O pressure increased by 74% in controls and 77% in obese subjects. Endothelium-independent vasodilation to SNP was not different between obese and control subjects in either SAT (Figure 7A) or VAT (Figure 7B) arterioles. Moreover, SNP-induced vasodilation in Hcy-preconditioned arterioles was mostly preserved and showed little reductions compared to those in unconditioned arterioles (Figure 7C,D).

**Figure 8:**
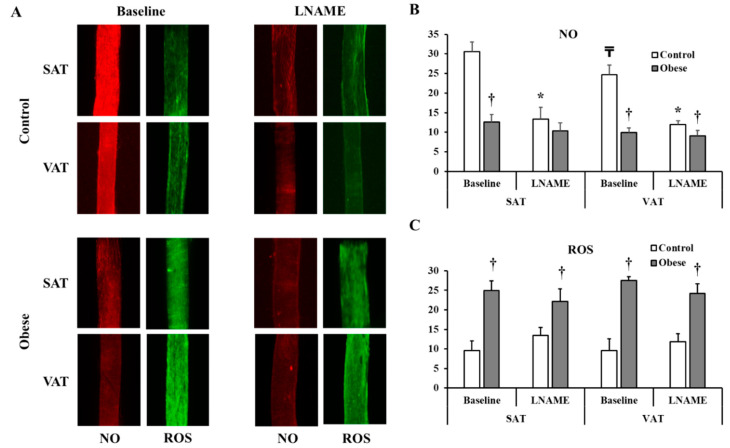
NO and Reactive Oxygen Species (ROS) production in isolated adipose tissue arterioles. (**A**): Representative images by fluorescence microscopy of NO (red fluorescence) and ROS (green fluorescence) generation at baseline conditions and after incubation with L-NAME in adipose tissue arterioles collected obese subjects (*n* = 40) and non-obese controls (*n* = 40). The charts present NO (**B**) and ROS (**C**) fluorescent signals that were measured and expressed in arbitrary units using NIH Image J software. All measures are represented as means± SE. * (*p* < 0.05) for comparing L-NAME to baseline in each group, † (*p* < 0.05) for comparing obese subjects with controls, and ₸ (*p* < 0.05) for comparing SAT and VAT arterioles in each treatment condition.

**Figure 9:**
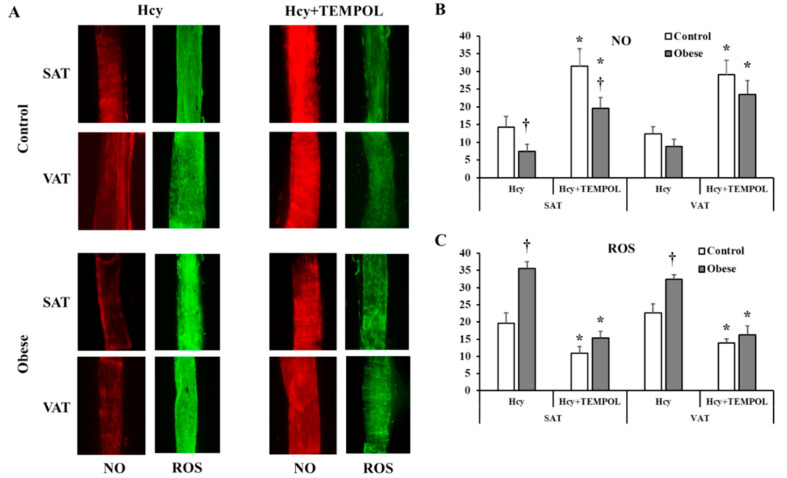
NO and ROS changes in response to homocysteine (Hcy) and TEMPOL. (**A**): Representative images by fluorescence microscopy of NO (red fluorescence) and ROS (green fluorescence) generation in response to Hcy and Hcy + TEMPOL treatment conditions in adipose tissue arterioles collected obese subjects (*n* = 10) and non-obese controls (*n* = 10). The charts present NO (**B**) and ROS (**C**) fluorescent signals that were measured and expressed in arbitrary units using NIH Image J software. All measures are represented as means± SE. * (*p* < 0.05) for comparing L-NAME to baseline in each group, and † (*p* < 0.05) for comparing obese subjects with controls.

Consistent with the FID data, arteriolar NO staining intensity (measured by ImageJ and expressed in arbitrary units) was lower in the obese compared to non-obese subjects in both SAT (obese: 12.6 ± 1.9, control: 30.6 ± 2.5, *p* < 0.0001) and VAT arterioles (obese: 9.9 ± 1.2, control: 24.7 ± 2.4, *p* < 0.0001) (Figure 8A,B). As opposed to NO, ROS staining was higher in obese subjects compared to controls in SAT (obese: 24.9 ± 2.6, control: 9.6 ± 2.5, *p* < 0.0001) and VAT arterioles (obese: 27.5 ± 1.0, control: 9.5 ± 3.1, *p* < 0.0001) (Figure 8A,C). Arteriolar NO was attenuated in response to L-NAME incubation in controls (% reduction in SAT = 56% and VAT = 51%, *p* < 0.0001) and obese subjects (% reduction in SAT = 18% and VAT = 9%, *p* > 0.05) (Figure 8). Similarly, Hcy incubation resulted in reductions in NO generation in controls (% reduction in SAT= 53% and VAT = 49%, *p* < 0.0001) and obese subjects (% reduction in SAT = 41% and VAT = 12%, *p* < 0.05) (Figure 9A,B). The L-NAME- and Hcy-mediated reductions in arteriolar NO were of a higher magnitude in controls compared to obese subjects and in SAT compared to VAT arterioles. This differential response could be attributed to higher baseline levels of NO in controls and SAT arterioles and accordingly, higher NO sensitivity. ROS staining increased in response to Hcy incubation in all participants (% increase in SAT = 104% and 43% and VAT = 137% and 38% in controls and obese, respectively) (Figure 9A,C). This induction in ROS was abolished in response to TEMPOL (% reduction in SAT = 45% and 56% and VAT = 39% and 50% in controls and obese, respectively). Reductions in ROS generation in response to TEMPOL was also associated with improvements in arteriolar NO staining (% increase in SAT = 1.2 and 1.6 folds and VAT = 1.4 and 17 folds in controls and obese, respectively) (Figure 9A,B).

Table 4 summarizes folate and vitamin B12 administration in obese and non-obese subjects. In subjects who administered supplements that contain folate or vitamin B12 before surgery, we observed no differences in arteriolar FID compared to those who were not taking supplementation. Moreover, the effect of L-NAME on inhibiting vasodilation in participants taking folate or vitamin B12 supplementation followed the same patterns observed in all participants. Data about alcohol consumption (frequency and quantity) were collected from all participants and summarized in Table 4. Using drinking level categories modified from those of Cahalan et al. [18], participants were classified as abstainers, light, moderate, and heavy drinkers. The criteria for this classification are shown under the table. Alcohol consumption was found to correlate negatively with folate levels (*r* = −0.367, *p* = 0.015), vitamin B12 concentrations (*r* = −0.207, *p* = 0.047), and SAT and VAT arteriolar FID at Δ60 cmH_2_O (*r* = −0.212, *p* = 0.035 and *r* = −0.206, *p* = 0.039, respectively).

## 4. Discussion

The main findings of the current study are that (1) plasma levels of Hcy were higher while folate, vitamin B12, and NO levels were lower in obese subjects compared to non-obese controls, (2) brachial artery FMD and arteriolar FID and AchID were lower in the obese compared to the non-obese subjects and were not affected by mild or moderate alcohol consumption or administration of vitamin B12 or folic acid, (3) sensitivity to eNOS inhibition via L-NAME was higher in the non-obese controls especially in the SAT-isolated arterioles, (4) exogenous Hcy incubation reduced arteriolar FID to a greater extent in the controls compared to obese subject (5) Hcy-preconditioned arterioles lost sensitivity to eNOS inhibition via L-NAME, and (6) endothelium-independent vasodilation was not significantly different between obese and non-obese subjects, and was mostly preserved after incubation with Hcy.

Hyperhomocysteinemia has been classified as mild (15–20 µmol/L), moderate (21–100 µmol/ L), and severe (>100 µmol/L). Nevertheless, there are also expected graded increased risks for subjects with Hcy concentrations of 10–15 µmol/L. In support of this, some epidemiological studies reported a higher risk of developing peripheral arterial disease and cardiovascular events in subjects with Hcy concentrations above 10 µmol/L [19,20,21]. In the current study, Hcy concentrations were not severely elevated in the obese group (11.4 ± 0.3 µmol/L) and might be considered within the normal range by some classifications. Nevertheless, Hcy levels were significantly higher in obese subjects compared to non-obese controls (8.7 ± 0.2 µmol/L) and correlated significantly with higher body weight, waist circumference, BMI, and fat percentage. Comparable concentrations of Hcy have been shown by Vaya et al. [22] in morbidly obese patients (12.76 ± 5.30 µmol/L) and normal-weight subjects (10.67 ± 2.50 µmol/L). In the latter study, obese patients had significantly higher Hcy levels than controls. Moreover, waist circumference and abdominal obesity were independent predictors of higher Hcy levels. Similarly, other clinical studies have reported concentrations of Hcy that ranged between 7 and 14 µmol/L in obese subjects [23,24,25,26]. Therefore, our data and results from previous studies may indicate that obesity-related hyperhomocysteinemia lies within the mild range.

Interestingly, within obese subjects, those with insulin resistance and hyperinsulinemia were shown to have higher levels of Hcy than obese insulin-sensitive individuals. For example, a study by Martos et al. [26] reported blood Hcy of 7.81 ± 0.52 µmol/L in obese hyperinsulinemic subjects versus 6.41 ± 0.17 µmol/L in obese normoinsulinemic (*p* = 0.002). Similar results were demonstrated by Sanchez-Margalet et al. [23]; average Hcy levels were 12.4 ± 0.5 µmol/L in obese hyperinsulinemic subjects versus 7.1 ± 0.7 µmol/L in obese normoinsulinemic subjects (*p* < 0.05). Our data are consistent with these findings and support the association between hyperinsulinemia and hyperhomocysteinemia in obese individuals. In the current study, we found higher Hcy concentrations in obese subjects with insulin levels more than 10 µU/mL (12.9 ± 0.2 µmol/L) compared with obese subjects with insulin levels less than 10 µU/mL (8.9 ± 0.4 µmol/L). Nevertheless, these correlations do not provide a clear mechanistic understanding of the relationship between elevated levels of insulin and Hcy and it is still unknown whether hyperhomocysteinemia is a consequence or a cause of hyperinsulinemia. Both conditions induce oxidative stress and they also exacerbate under oxidative stress, which could create a dangerous vicious circle.

Our results showed that plasma levels of Hcy correlate negatively with endothelial-dependent macrovascular (brachial artery FMD) and microvascular (arteriolar FID) function. Plasma Hcy was an independent predictor of vascular function even after accounting for other variables such as insulin, folate, and vitamin B12. Hyperhomocysteinemia was shown to be an independent risk factor for CVD. Several observational studies have reported positive associations between blood Hcy concentrations and CVD such as hypertension, stroke, coronary artery disease, and peripheral artery disease [27,28,29,30]. In a seminal meta-analysis by Boushey et al. [19], 27 observational studies that measured the link between Hcy levels in blood and CVD risk were included, and it was concluded that 5 μmol/L increments in plasma Hcy result in 1.6 to 1.8 fold increases in CVD risk. Endothelial dysfunction is one of the earliest signs of CVD. We have previously shown that endothelial-dependent vascular function and NO sensitivity are impaired in morbidly obese bariatric patients [14]. In these studies, we observed a role of vitamin D in improving endothelial-mediated FID in this category of morbidly obese patients, which could be achieved through the antioxidative properties of vitamin D. However, the reason behind the induction of oxidative stress in adipose tissue-isolated arterioles and whether it could be attributed to Hcy abundance is not entirely understood.

In the current study, we detected higher baseline levels of ROS and lower NO production in arterioles isolated from the non-obese group compared to obese subjects. Arteriolar levels of ROS correlated positively with plasma Hcy; however, this relationship does not imply causation. Thus, in an effort to determine the contribution of ROS production to Hcy-mediated vascular dysfunction in the current study, we measured ROS generation and FID in adipose tissue-isolated arterioles exogenously incubated in Hcy alone, and Hcy combined with the superoxide dismutase mimetic, TEMPOL. We observed significant induction in arteriolar ROS production, reduction in NO, and impairment in the FID after Hcy incubation; these changes were inhibited by the superoxide scavenger, TEMPOL. These findings may indicate a role of oxidative stress in mediating Hcy-induced endothelial dysfunction. Indeed, oxidative stress could be upstream to most of the previously suggested molecular mechanisms by which Hcy damages endothelial function. Some of these proposed mechanisms include NO inhibition, angiotensin II receptor-1 activation, prostanoid upregulation, and endothelin-1 induction [28,29,31,32,33]. These mechanisms were investigated in cultured endothelial cells or animal models of genetic- and diet-induced hyperhomocysteinemia. Thus, the current study provides some mechanistic understanding of Hcy-mediated microvascular dysfunction utilizing human isolated arterioles. However, further studies are required to elucidate molecular pathways involved in Hcy-induced oxidative stress such as NADPH and xanthine oxidases that could serve as therapeutic and preventive targets in Hcy-associated CVD.

In endothelial cells, oxidative stress and the resulting ROS are expected to interfere with NO bioavailability. Thus, it is anticipated that the major outcome of induced oxidative stress in endothelial cells is the interruption of NO production and, subsequently, NO-mediated vasodilation. Findings from our study supported this statement and demonstrated an abolishment of NO sensitivity in Hcy-treated vessels, as evidenced by a lack of any further impairment in the FID in response to L-NAME.

While endothelial-dependent FID was compromised in obese subjects and Hcy-preconditioned arterioles, endothelial-independent vasodilation (SNP-induced) showed little changes compared to controls and unconditioned arterioles, respectively. The response to endothelial independent vasodilators such as SNP in obese individuals has been inconsistent among different studies at both the micro- and macrovascular levels. For example, a study by Van Guilder et al. [34] reported no differences in forearm blood flow between lean and obese subjects in response to intra-arterial infusion of SNP while significant reductions in obese subjects compared to lean controls were reported by Schinzari et al. [35] using a similar approach. A similar discrepancy has been encountered for microvascular function. While in some studies, only endothelial-mediated vasodilation was different between lean and obese individuals with preservation of SNP-mediated vasodilation [36,37], other studies reported impairments in both endothelial-dependent and -independent vasodilation in obese subjects [38]. However, these microvascular studies were conducted on cutaneous microvessels, mainly capillaries, while in the current study we targeted adipose tissue-isolated arterioles. Regarding hyperhomocysteinemia, a study by Fu et al. [39] has shown that in a hyperhomocysteinemic rat model, both endothelial-dependent and -independent vasodilation of mesenteric arterioles were impaired. Nevertheless, a study by Schlaich et al. [40] reported an intact endothelial-independent forearm blood flow in subjects with elevated Hcy levels. Collectively, findings from the current study point to impaired NO-mediated vasodilation as the primary pathogenesis in vascular dysfunction that accompanies obesity and hyperhomocysteinemia. However, due to the existing inconsistency in the literature that investigates this topic, further studies are warranted.

A possible role of nutritional deficiencies of folate and vitamin B12 in the development of hyperhomocysteinemia has been suggested in previous studies. Randomized clinical trials have demonstrated that oral supplementation with a combination of folic acid and vitamins B6 and B12 lowered blood levels of homocysteine [19,41,42,43]. Furthermore, epidemiological studies showed that folate and vitamin B12 are major determinants of plasma homocysteine levels, and negative correlations have been reported between hyperhomocysteinemia and low levels of folate and vitamin B12 in patients with hypertension and coronary artery diseases [44,45,46,47]. In the current study, folate and vitamin B12 were found to be significantly lower in obese subjects compared to non-obese controls. Both vitamins correlated positively with each other and negatively with BMI, fat percentage, and parameters of glucose metabolism and insulin sensitivity. However, neither folate nor vitamin B12 correlated with blood levels of homocysteine in our cohort. Previous studies that investigated this association in the context of obesity have yielded inconsistent findings. While in some of these studies, an inverse correlation between Hcy concentrations and blood levels of folate and vitamin B12 have been found [46], other studies failed to prove this relationship [23,26]. This lack of evident correlations we, and some other studies, have encountered might indicate that blood concentration of Hcy reflects the collective contribution of several integrative factors and not only the folate and vitamin B12 status. These factors may include hyperinsulinemia [24], oxidative stress [48], inflammation [49], dietary habits [50], physical activity [51], alcohol consumption [52], metabolic function [53], or undiagnosed comorbidities.

Despite the lack of significant correlation with plasma levels of Hcy, folate and vitamin B12 were independent predictive factors of vascular reactivity and NO sensitivity in our participants. These findings are supported by previous clinical trials that demonstrated improvements in brachial artery FMD in response to folate or vitamin B12 supplementation in healthy people and patients with diabetes, coronary artery diseases, peripheral arterial occlusive disease, and hypercholesterolemia [54,55,56,57,58]. However, the current study is the first to report these associations regardless of folate or vitamin B12 intake. Moreover, it is the first study to investigate this association at the level of microvascular function using a unique ex vivo, tissue-isolated microvascular FID approach.

Previous studies suggested a role of increased alcohol consumption in inducing the risk of developing CVDs such as hypertension, stroke, coronary artery diseases, peripheral arterial diseases, and cardiomyopathy [59]. In our current study, we did not observe correlations between alcohol intake and systemic NO levels. However, we detected significant inverse associations between alcohol intake and endothelial-mediated vasodilation and NO sensitivity in adipose tissue-isolated arterioles. Moreover, alcohol inversely correlated with folate and vitamin B12 levels in plasma, which might be another mechanism by which it contributes to impaired vascular function. This assumption is supported by several studies showing that chronic alcohol exposure interferes with intestinal absorption, hepatic uptake, and renal conservation of folate [60]. It has also been reported that even moderate alcohol intake may reduce folate and vitamin B12 levels in healthy, well-nourished adults [61]. It was suggested by some studies that alcohol induces blood Hcy concentrations via its adverse effects on folate and vitamin B12 [52,62]; however, this assumption was rejected by other studies [63,64]. In our study, we did not find any correlation between alcohol intake and plasma Hcy concentrations; nevertheless, both predicted an impaired microvascular endothelial-dependent vasodilation. The relationship between alcohol consumption and plasma Hcy should be further explored in a larger number of subjects with more accurate quantification of alcohol intake and multivariate analysis of other possible factors that may modify Hcy.

There are some limitations to this study. First, we had a relatively small sample size, which carries out the risk of a type II error due to low statistical power. Second, in order to collect visceral adipose tissues, the non-obese controls had to be candidates for electives surgeries such as hernia and abdominal wall construction. These conditions may affect the general health of controls, and accordingly, the controls, despite being non-obese and devoid of any chronic disease, they are not considered healthy. Third, the obese group consisted of morbidly obese subjects who were considered for weight loss surgery, so these findings cannot be extrapolated to individuals who are mildly or moderately obese. Finally, one of the major limitations in our study is the unbalanced female to male ratio (1.1:1 in controls vs. 2.3:1 in the obese group) since fewer male patients are scheduled for bariatric surgery at our center (12.5%). Furthermore, most of the male candidates were excluded due to chronic comorbidities that may modify vascular outcomes. Although this study was not designed to determine gender-specific differences in the vascular response to different levels of Hcy, folate, vitamin B12, and alcohol, future studies are required to determine the influence of gender on vascular function.

## Figures and Tables

**Table 1:** Physical characteristics Cardiometabolic risk factors of study participants.

Variable	Non-Obese Controls	Obese Bariatric Patients	*p*-Value
*n*	40 (18 ♂)	40 (12 ♂)	
Age, y	35.4 ± 1.3	36.2 ± 1	0.339
Weight, kg	74.4 ± 1.6	142.4 ± 3.7 *	<0.001
BMI, kg/ m^2^	24.9 ± 0.5	50.6 ± 1.1 *	<0.001
WC, cm	131.5 ± 4	91.5 ± 2 *	<0.001
Body fat, %	32.2 ± 2.5	52.3 ± 1.0 *	<0.001
Body lean, %	65.3 ± 2.4	46.5 ± 0.9 *	<0.001
VAT mass, kg	0.7 ± 0.1	2.0 ± 0.2 *	0.0002
HR, bpm	74 ± 2	81 ± 1 *	0.004
SBP, mmHg	118 ± 2	132 ± 2 *	<0.001
DBP, mmHg	75 ± 1	80 ± 1 *	0.013
FPG, mg/dL	92 ± 2	103 ± 5	0.112
FPI, µU/mL	8.4 ± 1.4	14.9 ± 2.8 *	<0.001
HOMA-IR	1.9 ± 0.1	4.1 ± 0.4 *	0.002
HbA1c, %	5.3 ± 0.1	5.8 ± 0.2	0.148
Total chol, mg/dL	155 ± 9	165 ± 4	0.171
HDL, mg/dL	56 ± 6	43 ± 1 *	<0.001
LDL, mg/dL	87 ± 7	99 ± 4	0.116
TG, mg/dL	92 ± 11	115 ± 8	0.136
IL6, pg/mL	5.2 ± 0.7	21.6 ± 3.6 *	0.001
IL8, pg/mL	3.0 ± 0.1	4.9 ± 0.4 *	<0.001
CRP, mg/L	0.7 ± 0.1	3.8 ± 0.2 *	<0.0001

BMI, body mass index; Chol, cholesterol; cm, centimeters; CRP, C-reactive protein; DBP, diastolic blood pressure; FPG, fasting plasma glucose; FPI, fasting plasma insulin; HDL, high density lipoprotein; HOMA-IR, Homeostatic model assessment for insulin resistance; HR, heart rate; IL6, interleukin 6; IL8, interleukin 8; kg, kilograms; LDL, low density lipoprotein; n, number; SBP. Systolic blood pressure; TG, triglycerides; VAT, visceral adipose tissue; WC, waist circumference; y, years; ♂, males; * *p* < 0.05.

**Table 2:** Pearson correlations between subcutaneous (SAT) and visceral (VAT) arteriolar FID at Δ 60 and different physical and cardiometabolic variables.

	SAT Arteriole FID at Δ 60		VAT Arteriole FID at Δ 60	
	Pearson Correlation	*p* Value	Pearson Correlation	*p* Value
Weight	−0.856	<0.0001	−0.873	<0.0001
WC	−0.761	<0.0001	−0.761	<0.0001
BMI	−0.916	<0.0001	−0.937	<0.0001
Fat%	−0.839	<0.0001	−0.843	<0.0001
Lean%	0.833	<0.0001	0.837	<0.0001
VAT Mass	−0.625	<0.0001	−0.635	<0.0001
HR	−0.185	0.045	−0.187	0.043
SBP	−0.357	<0.0001	−0.355	<0.0001
DBP	−0.181	0.048	−0.172	0.057
FPI	−0.516	<0.0001	−0.500	<0.0001
HOMA-IR	−0.293	0.006	−0.289	0.006
HDL	0.350	0.004	0.373	0.002
Hcy	−0.427	0.003	−0.420	0.004
Folate	0.344	0.002	0.341	0.002
VitB12	0.432	<0.0001	0.421	<0.0001
Alcohol	−0.212	0.035	−0.206	0.039
FMD%	0.434	0.004	0. 349	0.017

**Table 3:** Pearson correlations between No sensitivity in SAT and VAT isolated arterioles and different physical and cardiometabolic variables.

% Reduction in FID at Δ 60 cmH_2_O Following L-NAME Incubation (NO Sensitivity)				
	SAT Arteriole		VAT Arteriole	
	Pearson Correlation	*p* Value	Pearson Correlation	*p* Value
Weight	−0.844	<0.0001	−0.428	<0.0001
WC	−0.649	<0.0001	−0.079	0.317
BMI	−0.914	<0.0001	−0.264	0.007
Fat%	−0.715	<0.0001	−0.173	0.126
Lean%	0.711	<0.0001	0.172	0.126
VAT Mass	−0.529	0.002	−0.051	0.399
SBP	−0.332	0.001	−0.220	0.021
HbA1c	−0.241	0.043	−0.326	0.009
FPI	−0.506	<0.0001	−0.363	0.001
HOMA-IR	−0.248	0.017	−0.266	0.011
HDL	0.385	0.002	0.392	0.001
Triglycerides	−0.245	0.036	−0.043	0.379
Hcy	−0.437	0.003	−0.298	0.033
NO	0.273	0.047	0.100	0.273
Folate	0.496	0.001	0.210	0.100
VitB12	0.435	<0.0001	0.231	0.029

**Table 4:** Consumption of alcohol and vitamin B12 and folate supplements.

**Supplement**	**Non-Obese Controls (*n* = 40)**	**Obese Bariatric Patients (*n* = 40)**
Folate (1 mg/d)	0 (0%)	3 (7.5%)
Vitamin B12 (250–5000 mcg/d)	0 (0%)	12 (30%)
**Alcohol**	**Non-Obese Controls (*n* = 40)**	**Obese Bariatric Patients (*n* = 40)**
Abstainers	25 (63%)	22 (55%)
Light drinkers	8 (20%)	13 (32.5%)
Moderate drinkers	7 (17%)	5 (12.5%)
Heavy drinkers	0 (0%)	0 (0%)

Light drinkers, less than one time/month with less than 5 drinks/time, 1–3 times/month with less than 3 drinks/time or 1–2 times/week with less than 2 drinks/time. Moderate drinkers, 1–3 times/month with 3–4 drinks/time, 1–2 times/week with 2–4 drinks/time, or 3–6 times/week with less than 2 drinks/time. Heavy drinkers, any quantity, and/or frequency that is more than moderate drinkers.
